# South Atlantic paleobathymetry since early Cretaceous

**DOI:** 10.1038/s41598-017-11959-7

**Published:** 2017-09-18

**Authors:** Lucía Pérez-Díaz, Graeme Eagles

**Affiliations:** 1 Royal Holloway University of London, TW20 0EX, Egham, Surrey, UK; 2 Alfred Wegener Institute, Helmholtz Centre for Polar and Marine Research, Am Alten Hafen 26, 27568 Bremerhaven, Germany

## Abstract

We present early Cretaceous to present paleobathymetric reconstructions and quantitative uncertainty estimates for the South Atlantic, offering a strong basis for studies of paleocirculation, paleoclimate and paleobiogeography. Circulation in an initially salty and anoxic ocean, restricted by the topography of the Falkland Plateau, Rio Grande Ridge and Walvis Rise, favoured deposition of thick evaporites in shallow water of the Brazilian-Angolan margins. This ceased as seafloor spreading propagated northwards, opening an equatorial gateway to shallow and intermediate circulation. This gateway, together with subsiding volcano-tectonic barriers would have played a key role in Late Cretaceous climate changes. Later deepening and widening of the South Atlantic, together with gateway opening at Drake Passage would lead, by mid-Miocene (∼15 Ma) to the establishment of modern-style thermohaline circulation.

## Introduction

A recent study^1^ suggests that the main control over ocean dynamics and, with it, paleoclimate and floral and faunal evolution, is imposed by changes in paleogeography. These controls are exerted in various ways, for example by the opening or closure of oceanic gateways or the uplift and subsidence of submarine ridges and plateaus. Reliable bathymetric models are thus important as datasets and boundary conditions for considerations of paleoceanography and paleoclimate.

The South Atlantic ocean, with its long history of growth, diverse topography of tectonic and magmatic ridges and plateaus, long continental margins, northern and southern gateways and extreme variations in sediment thickness, is a case in point. At present, it represents a clear pathway for the transport of deep water formed at both poles, but before this it developed important gateways at Drake Passage in the Cenozoic and across its equatorial reaches in the Cretaceous, and hosted a number of large igneous provinces at a variety of latitudes. Previous attempts at modelling South Atlantic paleobathymetry were done in sketch form or at low spatial resolution owing to the quality of the datasets available for tectonic reconstructions and limited knowledge about many of the processes that have contributed to the present-day shape of the South Atlantic. Sclater and McKenzie^2^ and later Sclater *et al*.^3^ present a number of sketches of South Atlantic paleobathymetry in which depths are dependent on seafloor age alone as defined by the rotation parameters of Bullard, Everett, and Smith^4^. They attempted to incorporate the Rio Grande and Walvis ridges, prominent submarine highs that formed as large igneous provinces, in anticipation of their important role in water circulation. More recently, Sykes *et al*.^5^ calculated sediment-free paleobathymetric reconstructions at 0.5 degrees resolution for the southern hemisphere using present-day bathymetry, sediment thickness and seafloor age grids. They were able to show acceptable correlations (∼500–700 m) with paleobathymetric estimates from a handful (6 in the South Atlantic) of DSDP and ODP drill sites. A further study by Hay *et al*. (1999)^6^ focuses on oceanic connections and their impact on climate for the Cretaceous period.

Müller *et al*.^7^ produced global paleobathymetry maps for oceanic regions that implemented corrections of the depth to a thermally-subsiding oceanic lithosphere for the presence of sediments and selected large igneous provinces, with continental locations through time determined using a mixture of visual fits and the results of statistical fitting procedures that provide a more detailed set of rotation parameters than Bullard *et al*.’s^4^.

Most recently, we have developed a workflow to improve on these results by using finer resolution (up to 1 arc-minute) input datasets, and by accounting for further processes including some of those involved during sedimentation, intraplate volcanism, subsidence of the continent-ocean transition zones, and convection of the mantle beneath the lithosphere^8^. By comparison of modelled to measured present-day bathymetry, Supplementary Figure FS01 shows that this workflow is likely to result in significantly more reliable paleobathymetric estimates than that of Müller *et al*.^7^. Beyond this, our reconstructions are built within the context of a recent high-resolution set of rotations that provide quantitatively the most reliable relative continental locations through time for the South Atlantic^9^. The differences between these and estimates in the older schemes used by other authors^2,3,5,7^ are significant at times prior to 84 Ma, for which Euler poles all lie considerably further south than those of Pérez-Díaz and Eagles^9^. Supplementary Figure FS02 illustrates the consequences of these differences, which most strongly affect the estimated opening of the central Atlantic gateway.

**Figure 1:**
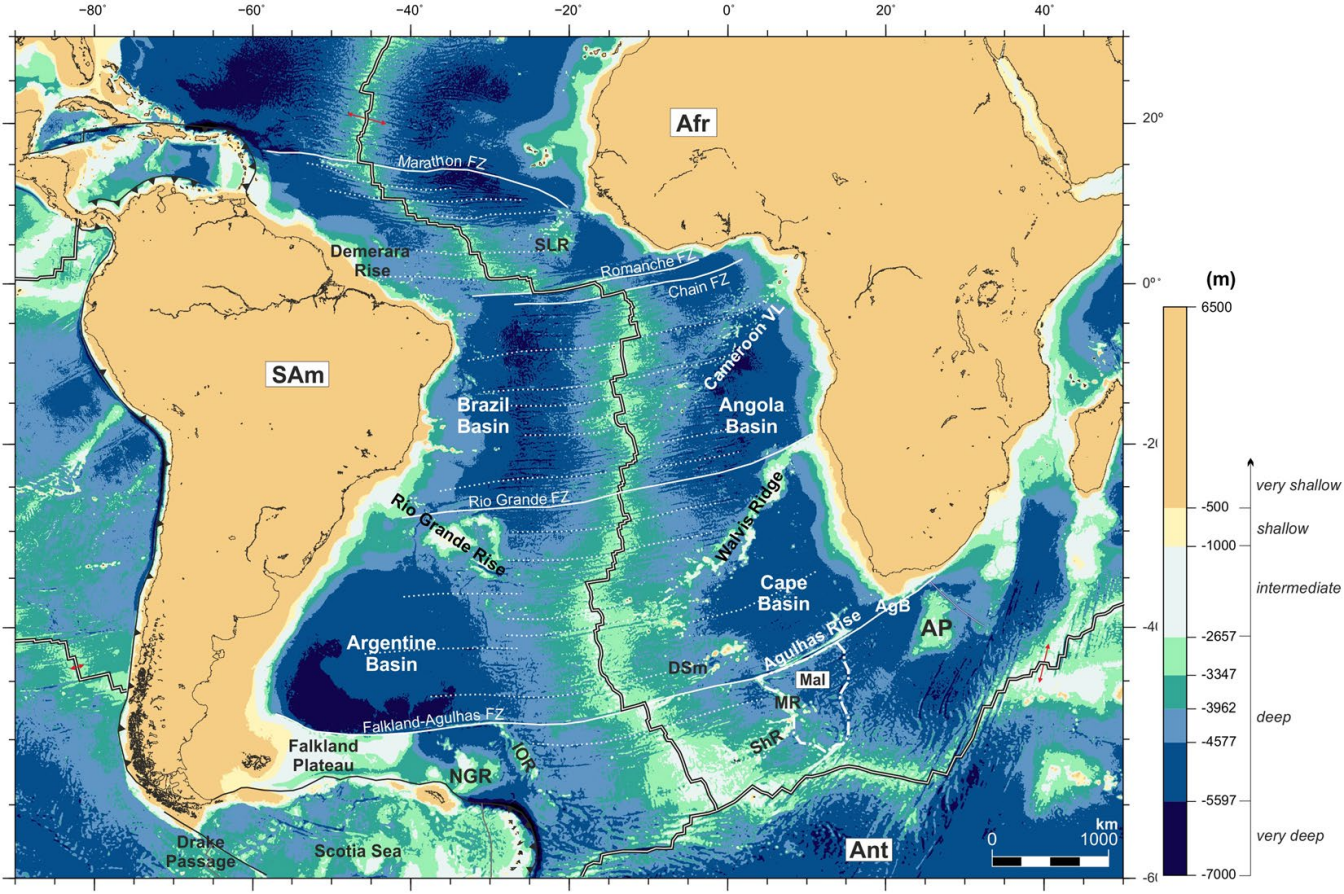
Satellite-derived present-day bathymetry^75^ and depth classes used for the paleobathymetric reconstructions shown throughout this section. Afr: African Plate; AgB: Agulhas Basin; Ant: Antarctic Plate; AP: Agulhas Plateau; Cameroon VL: Cameroon Volcanic Line; DSm: Discovery Seamounts; IOR: Islas Orcadas Rise; MR: Meteor Rise; NGR: North Georgia Rise; SAm: South American Plate; ShR; Shona Ridge; SLR: Sierra Leona Rise. The map was generated using the Generic Mapping Tools^76^.

Figure 1 shows satellite-derived present-day bathymetry assigned to eight depth classes. The models consist of a continuous range of depths across all of these classes, and so for other purposes could be displayed in different ways. The eight classes are suitable for describing and discussing the paleobathymetric reconstructions because they reflect a combination of paleoceanographic considerations and the tectonic considerations used to build them, as follows - four very deep classes (all <−5597 m) reflect the resolution of General Circulation Models describing the patterns of water movement and oceanic current structures within the world’s oceans^10^ within depths exceeding the maximum achievable in plate-cooling models for the oceanic lithosphere). The deep class (−5597 to −2657 m), encompasses depths in one of these plate cooling models, GDH1^11^. The intermediate depth class (−2657 to −1000 m) covers the depth range between the depth of the mid ocean ridge crests and a depth on most continental slopes. The shallow class (−1000 to −500 m) tends to follow strong gradients like those along the present-day continental slopes within which water-shelf interactions play a role. The very shallow class (<−500 m) lies within the range of expected minimum uncertainty in the reconstruction method^8^, and so might be taken as an estimate of uncertainty in the seawards locations of paleo-coastlines.

In discussing paleobathymetric reconstructions we will move forward in time, starting with the early stages of continent separation and maintaining the South American plate anchored in its present-day position.

## Methods and Uncertainties

**Figure 2:**
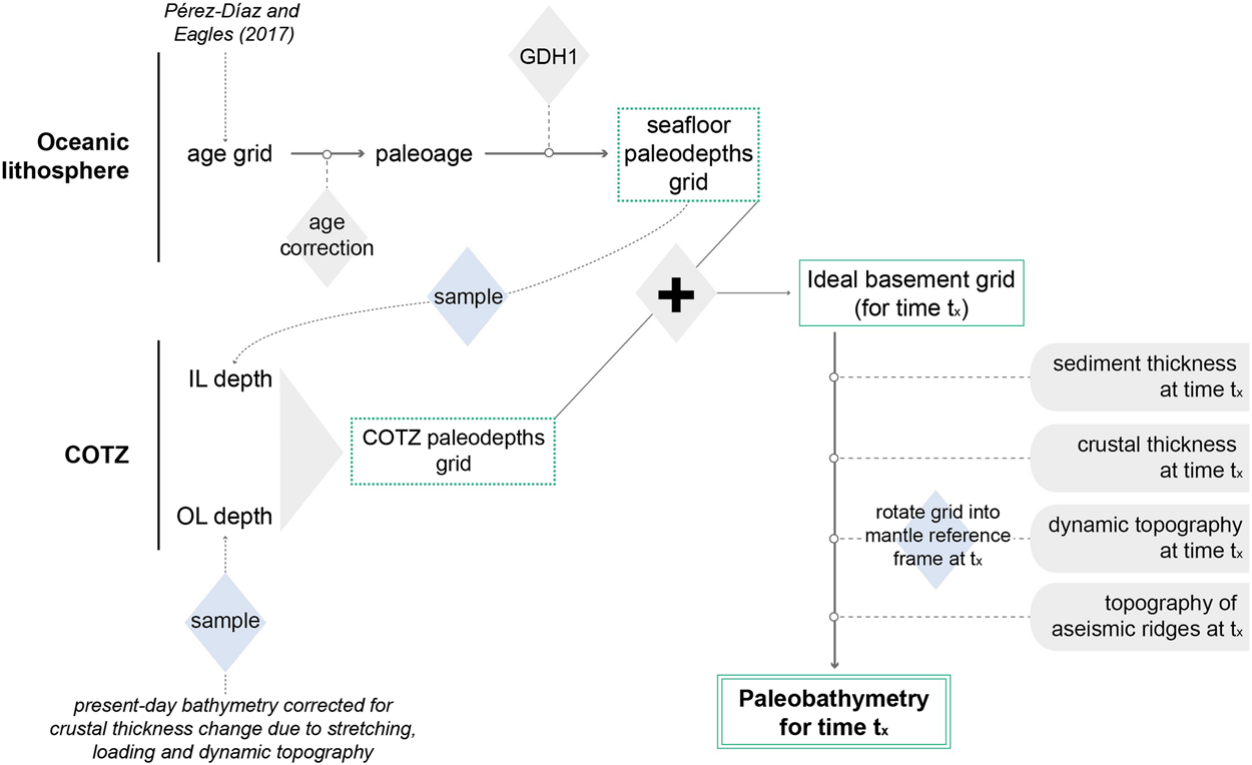
Summary of the workflow followed to produce paleobathymetric reconstructions for the South Atlantic.

Our method for modelling paleobathymetry and its uncertainties is described in detail elsewhere^8^. In less detail, we describe here and in Fig. 2 how the modelling procedure follows a number of steps. First, like Sykes *et al*.^5^ we calculate an idealised basement surface by modelling the subsidence of oceanic lithosphere as a function of its age. Here, we apply the GDH1^11^ plate cooling model to the high-resolution seafloor age grid of Pérez-Díaz and Eagles^12^. Although other thermal models may be preferable for other ocean basins, plate-cooling models in general and GDH1^11^ in particular adequately replicate the apparent subsidence profile in the South Atlantic. For most ages, GDH1^11^ predictions are within 100 m of the observed depths used to build it in the South Atlantic and discrepancies do not exceed 300 m in any location. For this reason it would be expected that uncertainties related to our choice of thermal model are small. A second source of uncertainty when modelling thermal subsidence arises from the choice of a particular age grid, and the possible errors within it. As well as being derived from a tightly constrained plate kinematic model, the seafloor age grid of Pérez-Díaz and Eagles^12^ includes a detailed uncertainty analysis.

When following our proposed workflow for modelling present-day bathymetry, GDH1^11^ is applied directly to the seafloor age grid of Pérez-Díaz and Eagles^12^. For times in the past it is necessary to first generate a correction surface that removes ages younger than the time of interest from the present-day seafloor age grid and reduces the remaining ages by the same amount to yield oceanic paleo-ages.

In order to achieve smooth paleobathymetric reconstructions, covering not only the oceanic parts of the South Atlantic but also its margins, we extend this thermally subsiding basement surface across the neighbouring continent-ocean transition zones. We do this by stretching the thermally subsiding basement surface between an undoubtedly-oceanic inner line and an outer line on supposedly non-extended continental crust. Depths along the inner line are defined as predicted by GDH1^11^ for the reconstruction’s paleo-age. Heights along the outer line are calculated by removing the effects of crustal thickness contrasts, post-breakup sedimentation, and dynamic topography on their present-day bathymetry. This approach relies on the assumption that the long-wavelength shape of the basement underlying continent-ocean transition zones is a result of extensional tectonics and breakup volcanism and therefore remains constant post-breakup. Because we do not attempt to palinspastically restore^13^ the extended continental margins, the assumption of stable post-breakup basement topography should not introduce large errors to our modelling method.

Having calculated an idealised basement surface by applying plate-cooling theory to seafloor ages and integrating the results with depths along the extended continental margins, we then refine this surface to account for the effects of other processes and properties that affect paleobathymetry. These are sedimentation, crustal thickness variations and intraplate volcanism, and so-called dynamic topography that occurs owing to transmission of vertical viscous stress from the convecting mantle.

Lastly, we reconstruct the plates’ paleopositions using published rotation parameters^9^.

Some of these refinements are relatively well understood and quantifiable, and so are their associated uncertainties, such as modelling thermal subsidence. Others, such as dynamic topography modelling, are more susceptible to errors due to the large number of assumptions they require and, in some cases, such as modelling of continent-ocean transition zones, uncertainties are much more difficult to quantify due to poor understanding of the processes shaping these areas or low-resolution data. After considering these in some detail, Pérez-Díaz and Eagles (2017b) conclude that paleobathymetry modelled following the method described here is likely to be least reliable over parts of large igneous provinces close to the times of their eruption, and most reliable within the oceanic interiors for Neogene time slices. From an analysis of the separations between the maximum and minimum plausible depths in all time slices, they suggest that the uncertainty range is not smaller than 500 m for any significant region at any time and for 95% of modelled nodes may lie within an asymmetrical confidence range spanning 1800 m. Given the asymmetry, we choose to portray the uncertainty as a pair of shallowest and deepest plausible bathymetries for each time slice.

## Results

### A salty and anoxic primitive ocean (138 to 100 Ma)

**Figure 3:**
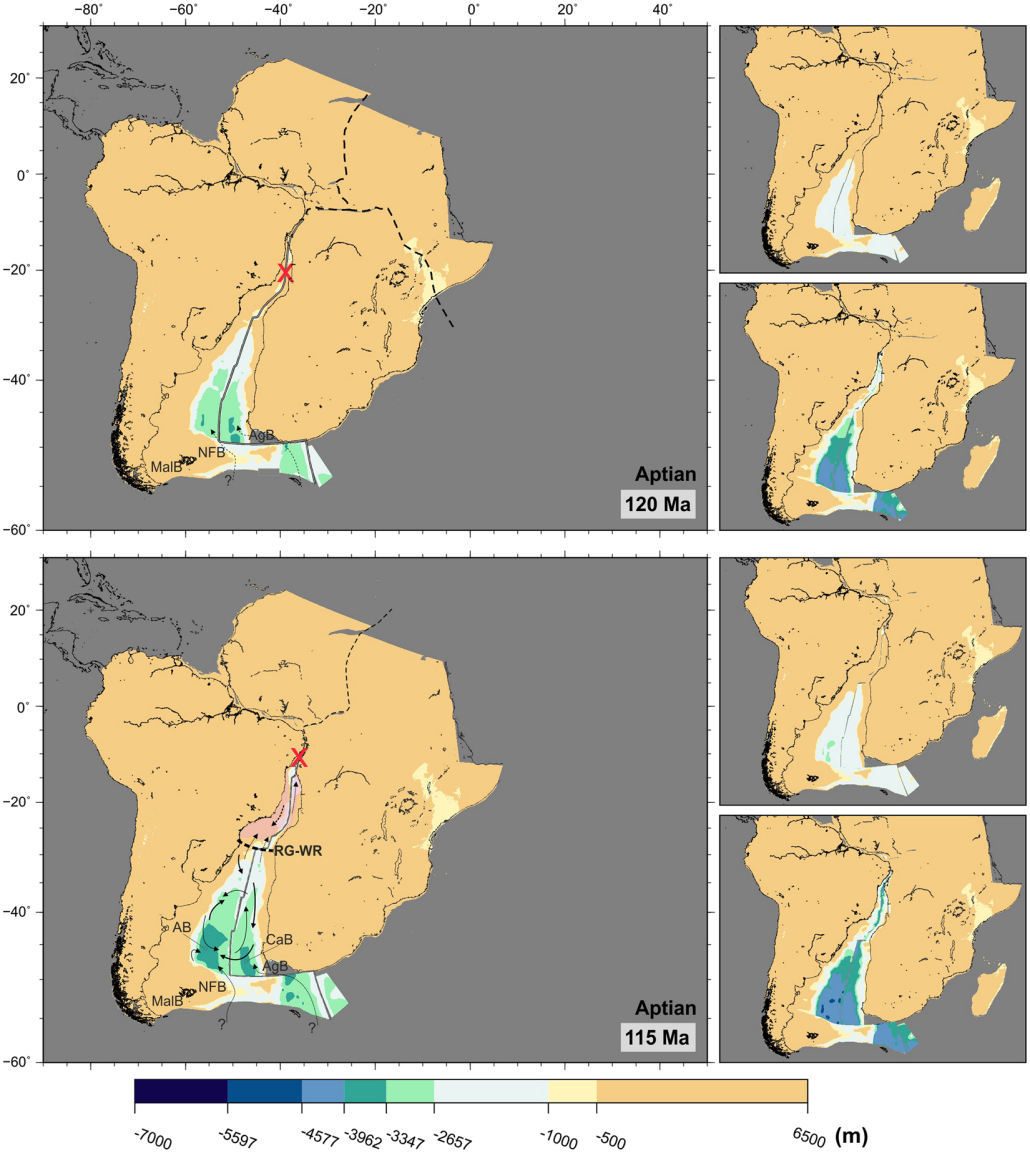
Paleobathymetric reconstructions at 120 and 115 Ma. Thick arrows: deep circulation; thin arrows: shallow circulation; dashed arrows: uncertain water circulation paths. Water circulation paths are qualitative and arrow length does not indicate current speed. Red ‘‘X”: gateways closed to significant water circulation; thick dashed lines: partial barriers to water circulation; pink shading: areas of salt deposition. Labels as before plus AgB: Agulhas Bank; AB: Argentine Basin; CaB: Cape Basin; MalB: Malvinas Basin; NFB: North Falkland Basin; RG-WR: Rio Grande Rise-Walvis Ridge. Panels next to the reconstructions show the shallowest (top) and deepest (bottom) paleobathymetries for the illustrated period by consideration of the uncertainties in the data and models used. The maps were generated using the Generic Mapping Tools^76^.

**Figure 4:**
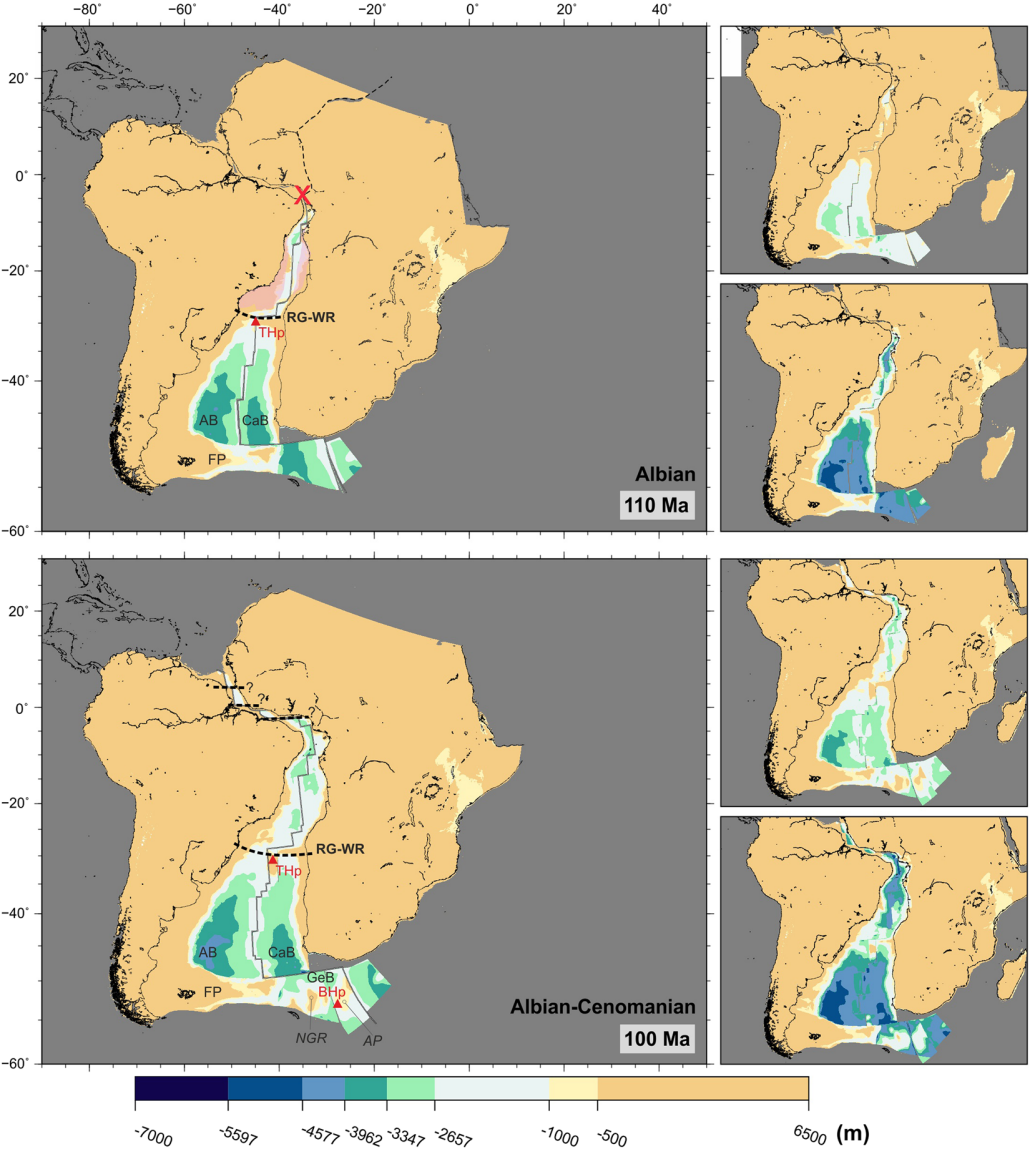
Paleobathymetric reconstructions at 110 and 100 Ma. Red ‘‘X”: gateways closed to significant water circulation; thick dashed line: partial barriers to water circulation; pink shading: areas of salt deposition. Labels as before plus BHp: Bouvet Hotspot; FP: Falkland Plateau; GeB: Georgia Basin; NGR: North-East Georgia Rise; THp: Tristán da Cunha Hotspot. This map was generated using the Generic Mapping Tools^76^. Panels next to the reconstructions show the shallowest (top) and deepest (bottom) paleobathymetries for the illustrated period by consideration of the uncertainties in the data and models used. The maps were generated using the Generic Mapping Tools^76^.

Figure 3 shows the initial stages of seafloor spreading between southern South America and South Africa, and the initial deepening of what later will become the Argentine and Cape basins. The southernmost part of these early basins reaches depths of over 3000 m within the first 20 My of seafloor spreading (bottom panel of Fig. 3). Figures 3 and 4 show the likelihood of an early shallow water connection to the young Southern Ocean over the Agulhas Bank during the very first stages of seafloor spreading. Water connections between the Southern Ocean and the southern South Atlantic via the Malvinas and North Falkland basins may have been possible at this time.

Figure 4 shows paleobathymetry at the time that seafloor spreading first reached the Gulf of Guinea (110–100 Ma). The first on-axis manifestations of the Tristan da Cunha hotspot show the early Rio Grande Rise-Walvis Ridge topography forming a barrier between the southern and northern regions of the South Atlantic. At 110 Ma, the restored Rio Grande Rise-Walvis Ridge topography mostly lies at depths shallower than 1000 m, but parts of the aseismic ridges deepen to 1500 m by 100 Ma. For times older than 100 Ma, no advection-based dynamic topography estimates exist owing to the difficulty of accounting for dispersion in the models of mantle circulation such estimates are based on. By comparison to present-day dynamic topography around active large igneous provinces^14^, it seems likely the effect of this would be to overestimate the depths of the Rio Grande-Walvis lineaments by as much as 1 km in reconstructions. We have corrected for such an overestimation conservatively by illustratively adding extra relief that smoothly varies to peak values of 500 m within a 500 km radius of the Walvis-Tristan hotspot location at times before 100 Ma. The barrier shown in Fig. 4 may therefore have been subaerially exposed along much of its length.

The bottom panel of Fig. 4 also shows seafloor spreading starting between the northern part of South America and West Africa and the incipient opening of an equatorial gateway. In the southernmost part of the South Atlantic, the Argentine Basin has reached depths of over 4000 m and is separated from the Cape Basin by a mid-ocean ridge which is some 2000 m shallower. As the Falkland Plateau clears the Cape region of Africa at 110–100 Ma, a new possibility for deeper connection between the Southern Ocean and the Cape Basin across the Georgia Basin becomes conceivable. This gateway is likely to have been shallower than shown, but still within the deep depth class, given its proximity to the likely dynamic topographic effects of the Bouvet mantle plume.

#### Implications for paleocirculation

Water circulation between the beginning of seafloor spreading at 138 Ma and the opening of gateways across the equatorial Atlantic and through the Georgia Basin at around 100 Ma would have been heavily controlled by the topography of the Falkland Plateau and that of the early Rio Grande Rise and Walvis Ridge. This makes it likely that, during early Cretaceous, the South Atlantic ocean circulated water in a set of isolated systems confined to individual basins, within which warm and salty water masses descended from shallow platforms towards the deeper domains to drive a “halokinetic” type of circulation^15^. Deep water sources within the Southern Ocean during the Albian have been suggested^16,17^ but at this point the transport of significant deep water masses into the South Atlantic across the Falkland Plateau is unlikely as it had not yet cleared the tip of Africa^9^.

By 115 Ma, seafloor spreading had progressed far enough north for the opening of a narrow basin between the coasts of Brazil and Angola (Fig. 3, pink shading). Limited connectivity at all but shallow depths across the Rio Grande-Walvis system would have allowed salinity to increase in this basin. Haline-driven convection triggered by excess evaporation would have resulted in the deposition of thick layers of salt, the timing and tectonic setting of which remain somewhat controversial^18–25^. Paleontological and sedimentological evidence suggest deposition occurred in water depths of no more than 500 m^26^. However, whether evaporites are part of the syn-rift sequence^19,20,22^ or postdate rifting^18,27^ is debated, as is their extent and continuity.

The controversies about timing, extent and depth of salt deposition overlap with the uncertainty estimates in our models. Seismic data and sedimentological studies will undoubtedly continue to play an important role in the debate. At face value, however, the preferred paleodepths modelled in Figs 3 and 4 tend to agree with a scenario of salt deposition in shallow (<2600 m) water during rifting. Transgression-regression events occurring over the Rio Grande-Walvis pair may have facilitated northern water intrusion into a warm and restricted environment with high water salinity and low oxygen content, favourable for the deposition of evaporites. Salt accumulation ceased by mid-Albian, with the improvement of circulation into the salt basin north of the Rio Grande-Walvis pair^21,23^. Cooler climatic conditions and greater depths south of the Rio Grande Rise- Walvis Ridge prevented the deposition of evaporites in the Argentine and Cape basins.

Further evidence of the anoxic conditions prevailing during the Aptian-Albian period lies in the large accumulations of organic-rich sediments^28–30^. Anoxic events in the South Atlantic have been linked to transgressive pulses inundating land areas and transporting terrestrial plant material seawards^31^. The distribution of dark-coloured sediments rich in organic matter (also known as sapropels) suggests that anoxic conditions periodically dominated much of the Cretaceous South Atlantic^30–32^.

### Opening of the equatorial Atlantic gateway (100 to 83 Ma)

**Figure 5:**
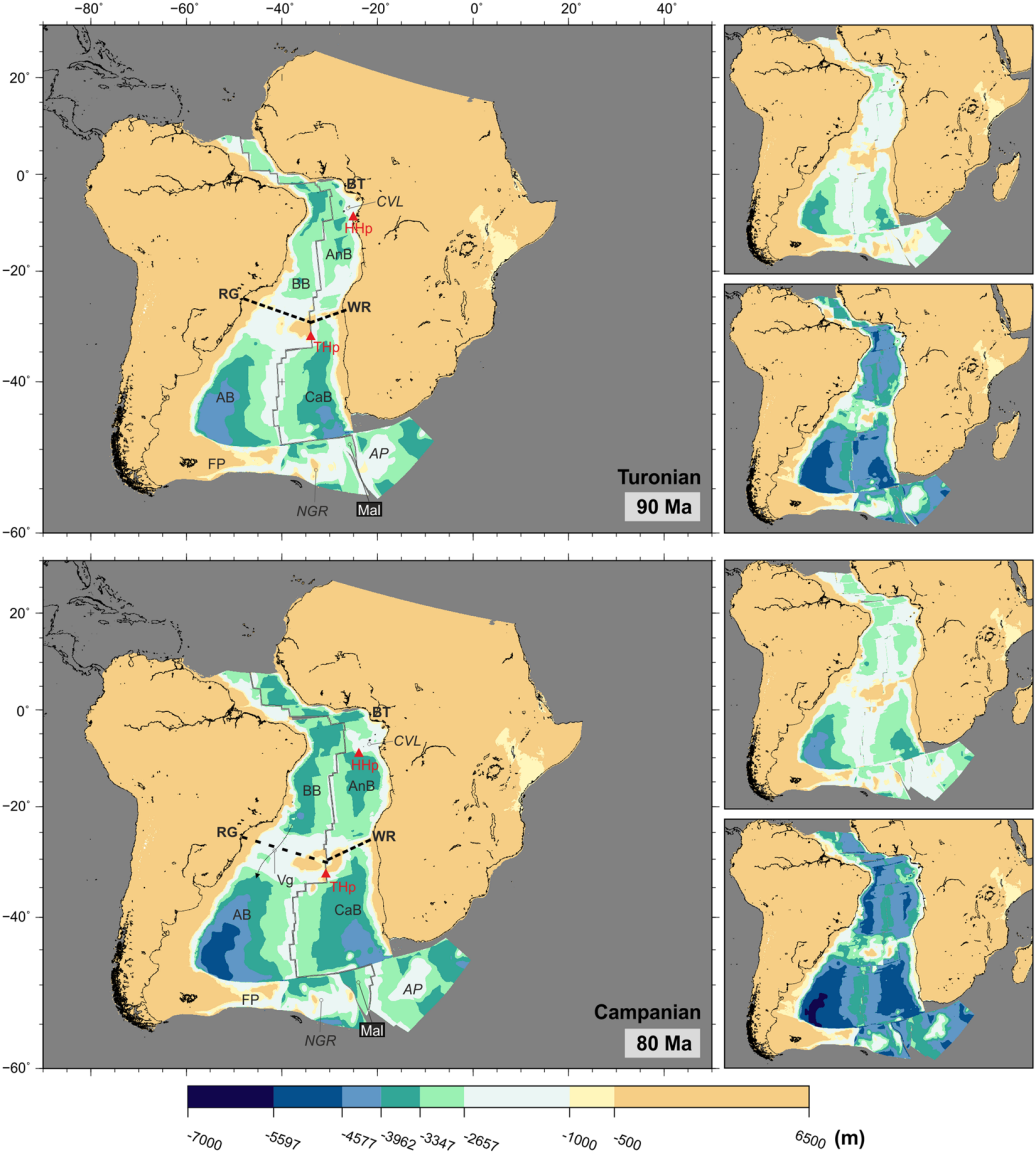
Paleobathymetric reconstructions at 90 and 80 Ma. Thick dashed lines: partial barriers to water circulation. Labels as before plus AnB: Angola Basin; BB: Brazil Basin; BT: Benue Trough; CVL: Cameroon Volcanic Line; HHp: St. Helena Hotspot; Mal: Malvinas plate; RG: Rio Grande Rise; V﻿g: Vema gap; WR: Walvis Ridge. Arrow length does not indicate current speed. Panels next to the reconstructions show the shallowest (top) and deepest (bottom) paleobathymetries for the illustrated period by consideration of the uncertainties in the data and models used. The maps were generated using the Generic Mapping Tools^76^.

A shallow (and possibly intermediate) water connection between the northern part of the South Atlantic and the Central Atlantic is likely to have been possible since 100 Ma. However, the fact that this connection started to form as a result of relative plate motions along continent-continent and continent-ocean transform boundaries, means that deeper water is likely to have been limited to the interiors of unconnected pull-apart basins separated by structural highs until the Turonian (90 Ma). The widening and deepening of the space between the northern South Atlantic and the Central Atlantic led to a fully open gateway by the start of the Campanian (84 Ma), with depths reaching 3500 m along the northern parts of the African and South American margins (bottom panel, Fig. 5).

In the Gulf of Guinea, the activity of the St. Helena hotspot leads to formation of visible topography in the reconstructions at times after 90 Ma (Fig. 5). In contrast, the Rio Grande-Walvis aseismic ridge had started developing well before this time, and by the start of the Campanian (84 Ma) a large portion of it had not yet subsided below 1500 m. However, within this pattern, a deeper channel had started forming across Rio Grande Rise, reaching depths of around 2000 m by mid-Campanian (80 Ma)(Fig. 5).

Further south, deepening of the Argentine and Cape basins continued, with depths reaching 5000 m in the Argentine Basin by mid-Campanian (80 Ma) (Fig. 5). As a result of the westward ridge jump to the Malvinas-Africa spreading centre at 97 Ma^9^, the topography of the North East Georgia Rise and Agulhas Plateau started to subside and increase the range of possibilities for N-S exchange between the South Atlantic and Southern oceans.

#### Implications for paleocirculation

The Albian-Cenomanian unconformity, present through most of the South Atlantic, may be a representation of the changes in water circulation that started at 100 Ma as a result of the opening of an equatorial gateway^30^.

The timings proposed by these reconstructions for the opening of a gateway between the northern South Atlantic and the Central Atlantic agree well with sedimentological, geochemical and faunal evidence gathered during oceanic drilling campaigns. These studies point to shallow water connections in the equatorial Atlantic starting in Albian times (113–100 Ma), and fully open oceanic conditions being partially established at some time between the Turonian and Campanian (94–72 Ma)^33–36^ or perhaps as late as the Maastrichtian (72–66 Ma)^16,37,38^.

The opening of the equatorial gateway, even before deep water exchange was possible across it and despite the limitation in north-south connection imposed by the Rio Grande-Walvis system, may have initiated a change in circulation patterns for the Brazil and Angola basins, from very restricted conditions in the early Cretaceous to more productive environments by the end of the period. However, it should be noted that surface marine water circulation into the Brazil and Angola basin at this time may have not occurred exclusively through the newly open passage with the Central Atlantic, but also during highstands from the Tethys Sea via the intracontinental Benue Trough, Niger basin and Sahara^30,39,40^.

**Figure 6:**
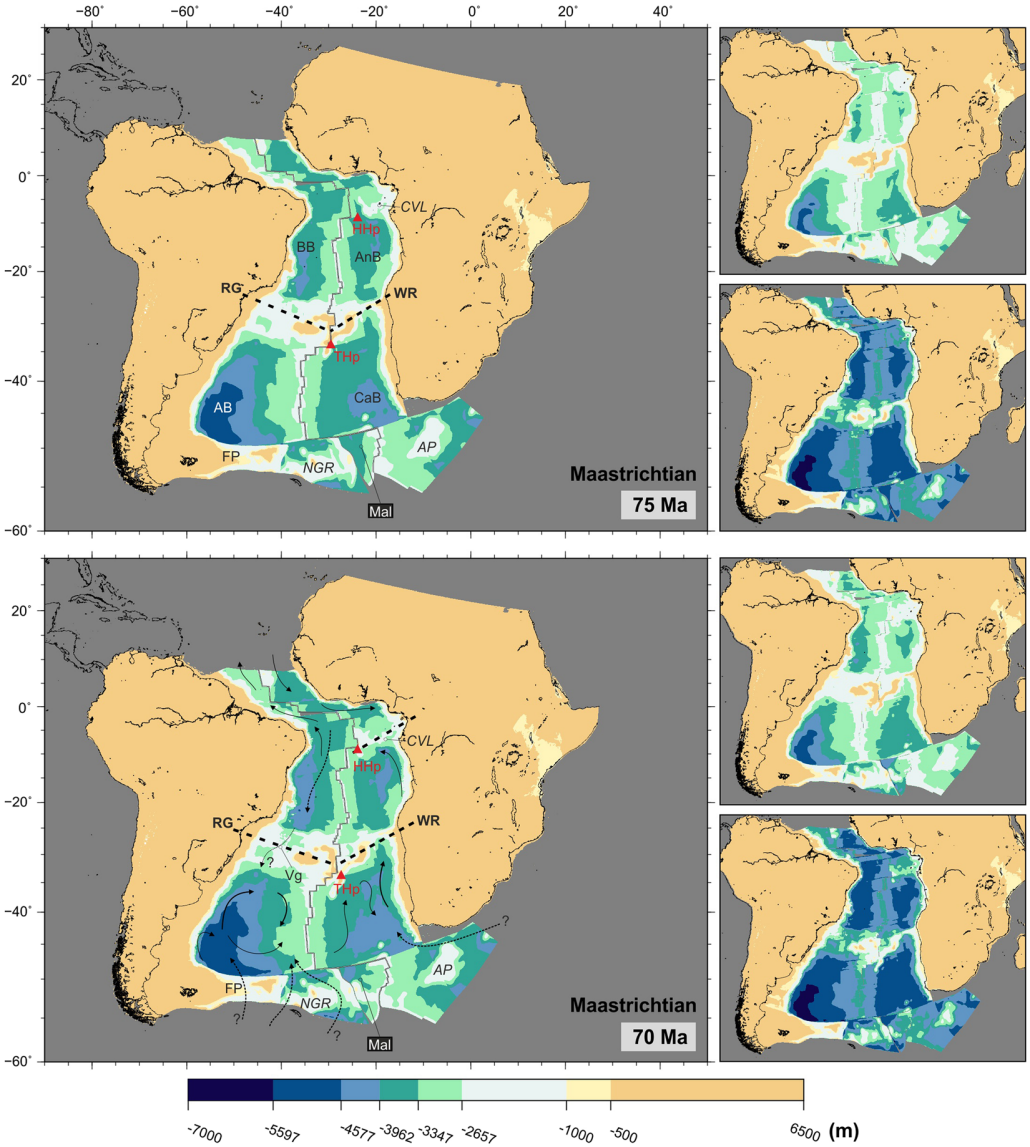
Paleobathymetric reconstructions at 75 and 70 Ma. Thick dashed lines: partial barriers to water circulation. Water circulation paths are qualitative and arrow length does not indicate current speed. Labels as before plus Vg: Vema Gap. Panels next to the reconstructions show the shallowest (top) and deepest (bottom) paleobathymetries for the illustrated period by consideration of the uncertainties in the data and models used. The maps were generated using the Generic Mapping Tools^76^.

Despite these newly established water connections, the presence of Coniacian (90–86 Ma) sapropels in the Angola basin speak to the periodic absence of oxygenated bottom waters at this time^28,30^. Prevailing isolated conditions in the Angola basin are likely to be the result of the barriers to water circulation imposed by the Cameroon Volcanic Line, Walvis Ridge, mid-ocean ridge and African margin, which are shown on reconstructions bounding the Angola basin at least until the end of the Cretaceous (Figs 5 and 6).

The Rio Grande-Walvis system continued to play an important role in restricting transport of water into the Argentine and Cape Basins via the subsiding Falkland Plateau and the circulation of the newly established shallow-intermediate water into the Brazil-Angola basins. Similarly as in early Cretaceous, the formation and circulation of most deep-water masses would still occur individually in restricted, relatively small oceanic basins^36,38,41,42^. Zimmerman *et al*.^30^ suggest minor water circulation from the Brazil Basin into the Argentine Basin via the Vema Gap (sometimes referred to in the literature as Rio Grande Gap)^43^ may have been possible after an Aptian-Albian spreading centre jump. Evidence for the occurrence of a ridge jump to form the Vema Gap, is also identified by Pérez-Díaz and Eagles^9^ but at the Cenomanian-Turonian boundary (93 Ma). In Fig. 5, the Vema Gap doesn’t exceed 2000 m in depth suggesting that, if north to south water circulation was happening at this time, it would have been limited to the exchange of relatively shallow water masses only.

### Late Cretaceous ocean-climate changes (83 to 66 Ma)

Connections between the Brazil and Argentine basins over the Rio Grande Rise continued improving during the Campanian-Maastrichtian (83–66 Ma, Figs 5 and 6). In addition to the Vema Gap, the Hunter Gap (sometimes Hunter Channel in the literature) started opening between the eastern Rio Grande Rise and the mid-ocean ridge. By the end of the Maastrichtian (66 Ma) the Vema Gap entered the deep water class, allowing for complete intermediate water connection. The Hunter Channel remained shallower than 2000 m at this time. Conversely, the eastern South Atlantic basins remained largely restricted during the late part of the Cretaceous. The Cape Basin, although well connected to the Southern Ocean, was separated from the Argentine Basin by the mid-ocean ridge and from the Angola Basin by the Walvis Ridge. The Angola basin remained confined on three sides by the Walvis Ridge, the mid-ocean ridge and the African margin, and water circulation from the north was still partly restricted due to topography formed along the St Helena hotspot trail, across which only narrow deep water channels existed by the end of the Cretaceous (Fig. 6).

From the early Campanian (80 Ma, bottom panel of Fig. 5), the opening of the South Atlantic had progressed enough for full intermediate water connections between all the major oceanic basins to be established. However, even if deep water formation at this time was occurring it seems unlikely that it would have driven global ocean circulation via South Atlantic pathways due to the barriers to deep water exchange imposed by the features reviewed above.

#### Implications for paleocirculation

The late Cretaceous was a period of decreasing global temperatures, from peak warmth in the Turonian (94–90 Ma) to cooler conditions at the end of the Maastrichtian^17,35,38,44–48^. These climatic changes and the changes in circulation patterns during the latest Cretaceous are recorded by stable isotope, sedimentological and faunal studies which evidence a reduction in the frequency of anoxic events, an increase in carbonate preservation and the partial disappearance of interbasin water differentiation by the end of the Mesozoic^16,17,37,47,49^. However, the feedbacks between the decreasing paleotemperatures, changes in circulation and the generation of deep-water masses are still poorly understood^15,16,37,50^.

Ocean-climate changes starting at the end of the Cretaceous would eventually lead to the establishment of thermohaline circulation as we know it today^1,36,38,42,51^. The opening of gateways across the South Atlantic during the Campanian (84–72 Ma) (Fig. 6) may have initiated Southern Component Water formation in the oceans of the southern hemisphere and its northward circulation towards the North Atlantic across the Central Atlantic Seaway^1,17,51–55^. Deep water masses may also have been forming in the high latitudes of the North Atlantic (Northern Component Water, NCW^38,42^, and North Pacific^1,47,56^.

**Figure 7:**
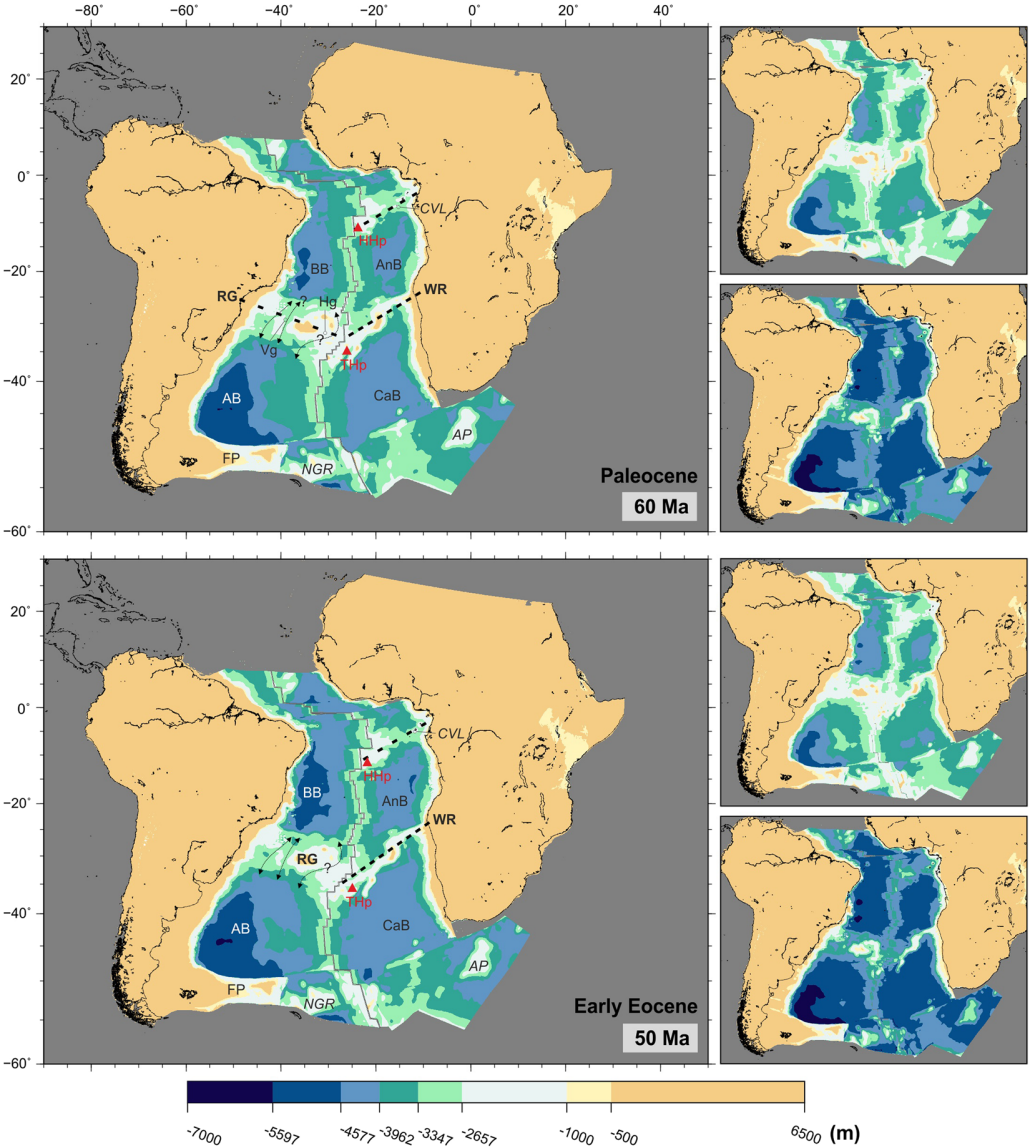
Paleobathymetric reconstructions at 60 and 50 Ma. Thick dashed lines: partial barriers to water circulation. Water circulation paths are qualitative and arrow length does not indicate current speed. Labels as before plus Hg: Hunter Gap. Panels next to the reconstructions show the shallowest (top) and deepest (bottom) paleobathymetries for the illustrated period by consideration of the uncertainties in the data and models used. The maps were generated using the Generic Mapping Tools^76^.

Subsidence of the Rio Grande-Walvis pair of lineaments has been variously suggested to have allowed deep-water circulation north towards the North Atlantic and across the Central African Seaway since early Campanian (80 Ma)^51,55^, Maastrichtian (71–66 Ma)^1,36,47^ or even mid-Paleocene times (60 Ma)^57^. This latest opening time is most consistent with the paleobathymetric reconstructions presented here (Fig. 7).

### Paleogene to present-day (66 to 0 Ma)

**Figure 8:**
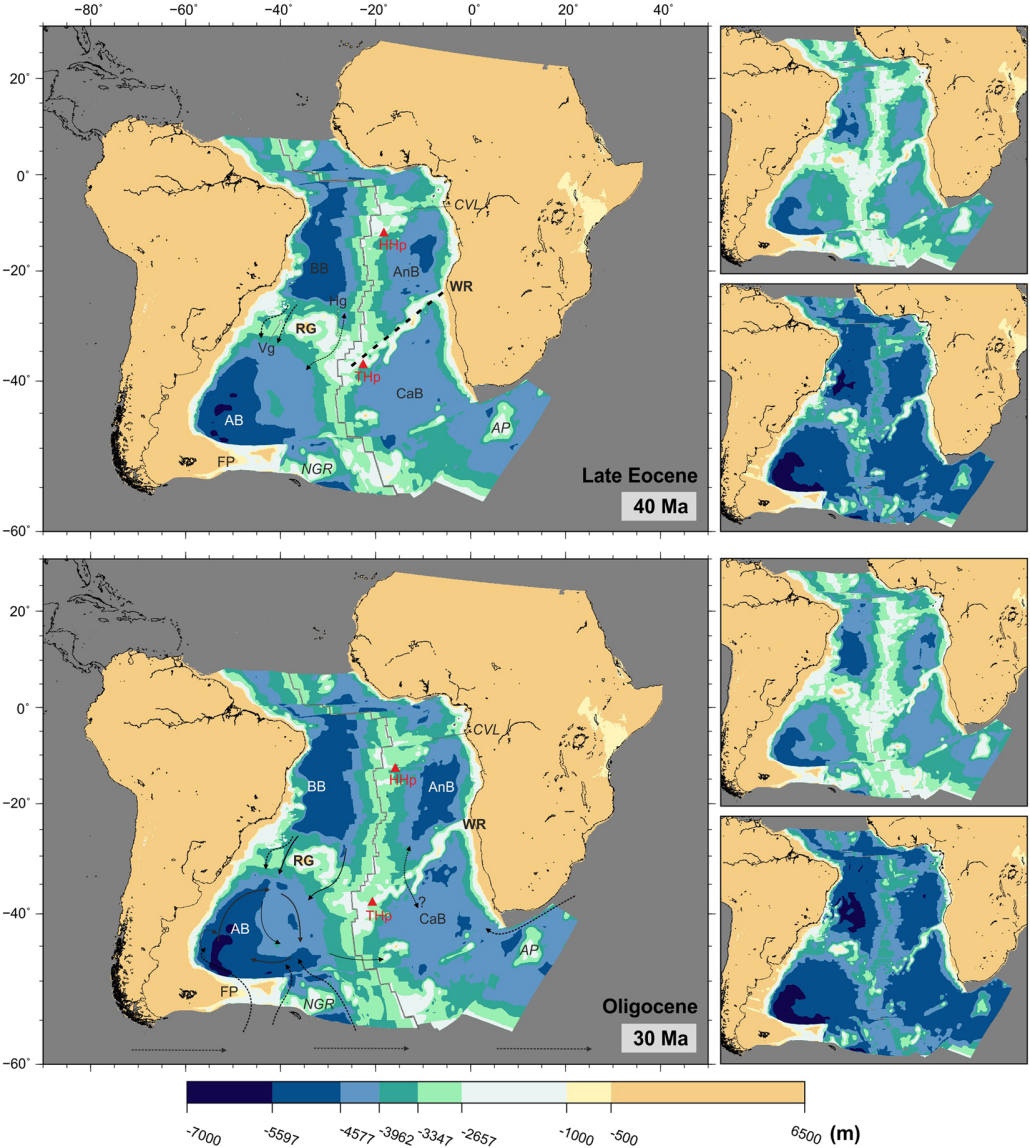
Paleobathymetric reconstructions at 40 and 30 Ma. Thick dashed lines: partial barriers to water circulation. Water circulation paths are qualitative and arrow length does not indicate current speed. Labels as before. Panels next to the reconstructions show the shallowest (top) and deepest (bottom) paleobathymetries for the illustrated period by consideration of the uncertainties in the data and models used. The maps were generated using the Generic Mapping Tools^76^.

The South Atlantic continued to deepen and widen, with the Argentine and Brazil basins reaching depths of over 5500 m by the start of the Eocene (Fig. 7). The Rio Grande and Walvis pair of lineaments continued to subside. The Vema Gap reached depths of 3500 m and together with the Hunter Gap (which remained some 500 m shallower) allowed exchange of all but bottom waters by the end of the Eocene (Fig. 8). A small part of the Rio Grande Rise remained at or near emergence until the mid-Eocene (40 Ma). The Paleogene opening of gaps through the Rio Grande Rise, together with a wider and deeper equatorial gateway and a subsiding Cameroon Volcanic Line would have contributed to improved ventilation of the Brazil and Angola Basins.

**Figure 9:**
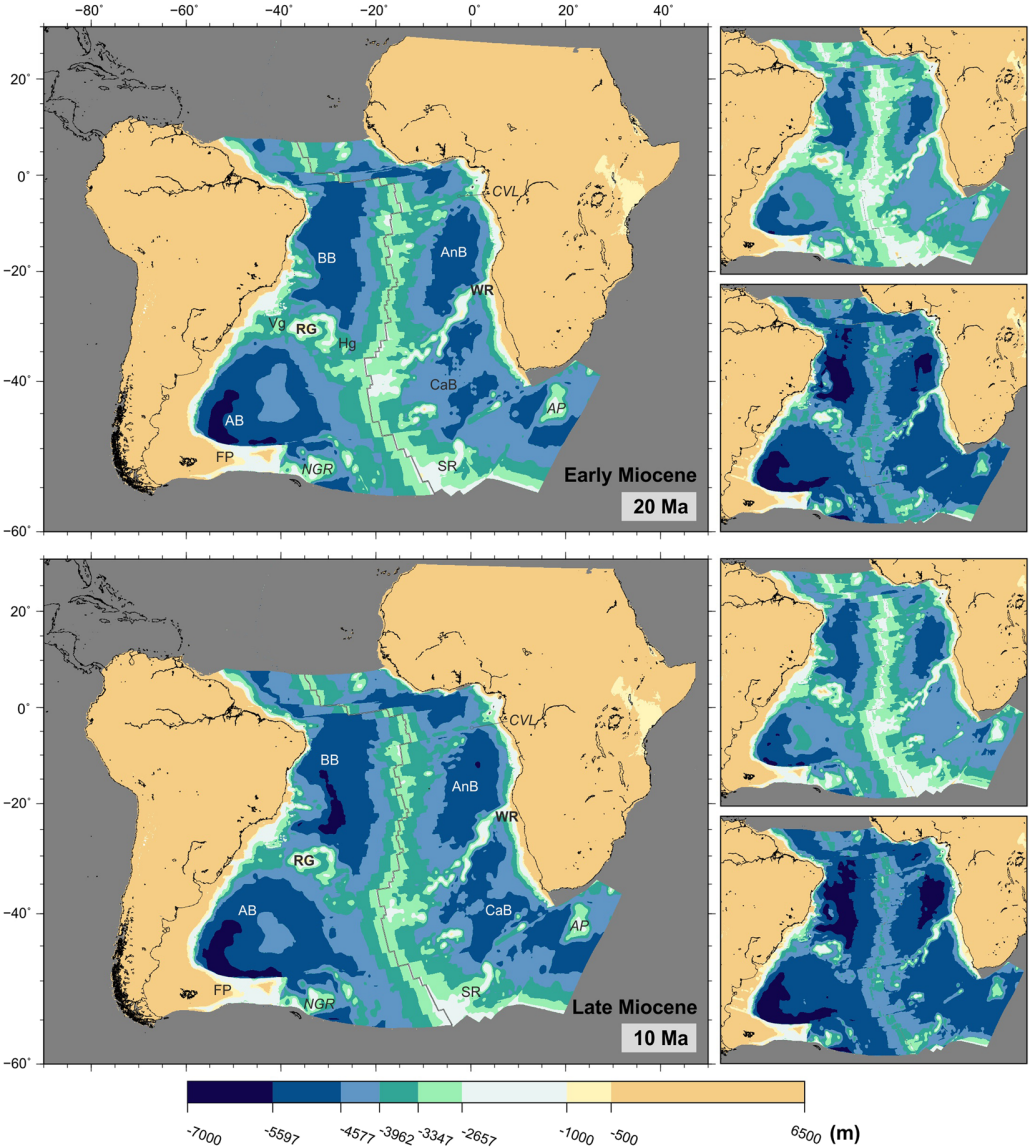
Paleobathymetric reconstructions at 20 and 10 Ma. Labels as before plus SR: Shona Ridge. Panels next to the reconstructions show the shallowest (top) and deepest (bottom) paleobathymetries for the illustrated period by consideration of the uncertainties in the data and models used. The maps were generated using the Generic Mapping Tools^76^.

Water circulation across the Walvis Ridge remained limited until mid-Eocene (40 Ma), when narrow channels between its Paleogene seamounts reached depths of 3000 m (Fig. 8, top panel). By the start of the Neogene (23 Ma), two gaps started deepening across the eastern part of the Walvis Ridge (Fig. 9, top panel).

Modelled Neogene paleobathymetry shows many similarities with the present-day South Atlantic. The mid-ocean ridge and Rio Grande-Walvis system separate four deep distinct basins, the Brazil, Angola, Argentine and Cape basins. The Cameroon Volcanic Line has stopped being a barrier to water circulation and gaps also exist across the Rio Grande Rise and Walvis Ridge. These gaps would have allowed the circulation of bottom water between the Argentine and Brazil basins since the mid-Miocene (15 Ma), when they reached depths of over 4000 m. No such connection existed between the Cape and Angola basins across the Walvis Ridge. Even at present-day, only a narrow gap deeper than 3000 m exists across the eastern part of the Walvis Ridge (Fig. 1, which is suggested by reconstructions to remain a partial barrier to deep water circulation between the Cape and Angola Basins.

#### Implications for paleocirculation

The changes in circulation trends and warm climate continue during the Paleocene. Although it has been suggested that interbasin connections between the Cape and Angola basins through theWalvis Ridge may also have become possible during the Paleocene^29^ this seems unlikely from the reconstructions shown in Fig. 7. Studies of the isotopic composition of sediments suggest that, until the start of the Oligocene (33 Ma), the entire water column at sites near the Walvis Ridge was occupied by a single water mass that originated from the Southern Ocean^58^. This is consistent with the reconstructions presented here, which show the Walvis Ridge as a barrier to significant water circulation until the Neogene (23 Ma).

In the late Eocene, with the onset of ice-sheet formation in Antarctica, formation of the first Antarctic cold deep waters started (proto-Antarctic Bottom Water, pAABW)^59^. This water mass may have circulated following paths similar to those observed for today’s Antarctic Bottom Water but its density contrast with other water masses would have been less and so it would have circulated at lower velocities^28^. Deep currents of this pAABW entered the South Atlantic from the south, possibly forming two independent gyres in the Argentine and Cape basins, but were unable to penetrate north of the Rio Grande-Walvis complex whose deepest channels were still shallower than 3500 m (Fig. 8).

Expansion of the Antarctic ice sheet continued throughout the Eocene and Oligocene, with formation of large ice sheets over the whole of Antarctica starting at 34 Ma^60,61^. An effective ocean gateway to circulation of Southern Ocean water may have developed across the Scotia Sea during middle Eocene times. This would have contributed to Eocene cooling and the early growth of Antarctic ice sheets^62^. However, larger changes in circulation leading to the formation of the Antarctic ice cap were the result of two events that took place during the Oligocene: the full opening of the Drake Passage^62–64^ and the separation of Australia from Antarctica and formation of the Tasman Strait^65,66^. Also during the Oligocene, significant production of North Atlantic Deep Water may have started^58^.

Separation of the Antarctic Peninsula from South America and opening of the Drake Passage to deep water exchange is estimated at 33–30 Ma, although deep water flow may have been restricted until 29 Ma at least^64,65,67^. A similar early Oligocene time has been proposed for the establishment of a strong current across the Tasman Strait between Australia and Antarctica^65,66,68^. Although Tasmania cleared East Antarctica much sooner (50 Ma), there is no suggestion that a seaway across the Tasman Strait existed until about 32–30 Ma^65,66^. These two events triggered a drastic change in oceanic circulation in the southern hemisphere. The Antarctic Circumpolar Current was established, limiting heat transfer between Antarctica and the lower latitudes and therefore favouring the generation of large ice sheets in Antarctica, in turn leading to the formation and circulation of Antarctic Bottom Water as we know it today by mid-Miocene. By this time, some bottom water circulation into the Brazil basin from the Argentine basin started to be possible (Fig. 9). Even today, only a small part of the Antarctic Bottom Water is able to pass across Rio Grande Rise into the northern part of the South Atlantic^43,69,70^.

**Figure 10:**
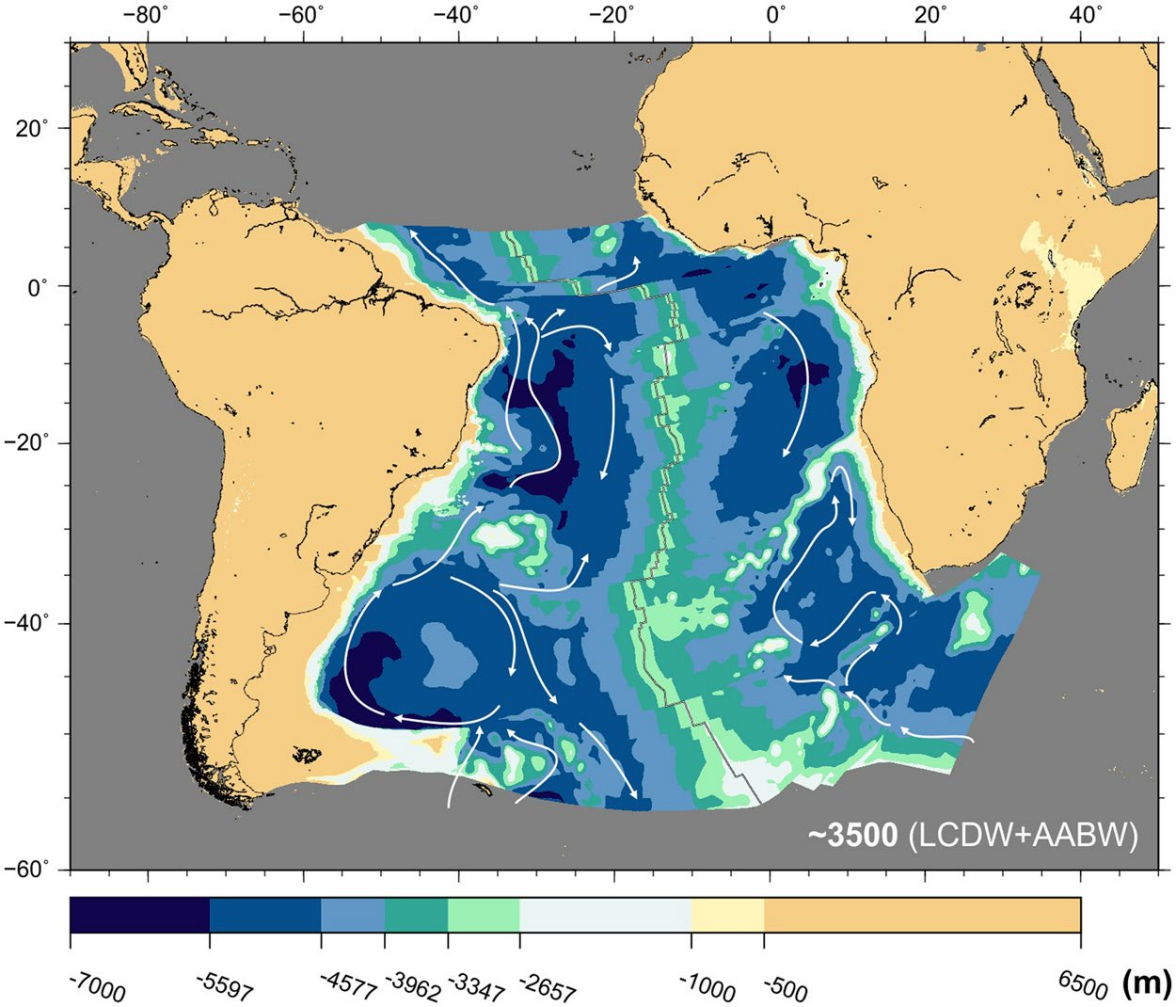
Modelled bathymetry at present-day and simplified patterns of bottom and deep water circulation (LCDW + AABW water masses). Water circulation paths are qualitative and arrow length does not indicate current speed. AABW: Antarctic Bottom Water; LCDW: Lower Circumpolar Deep Water^77–80^. Panels next to the reconstructions show the shallowest (top) and deepest (bottom) paleobathymetries for the illustrated period by consideration of the uncertainties in the data and models used. The maps were generated using the Generic Mapping Tools^76^.

**Figure 11:**
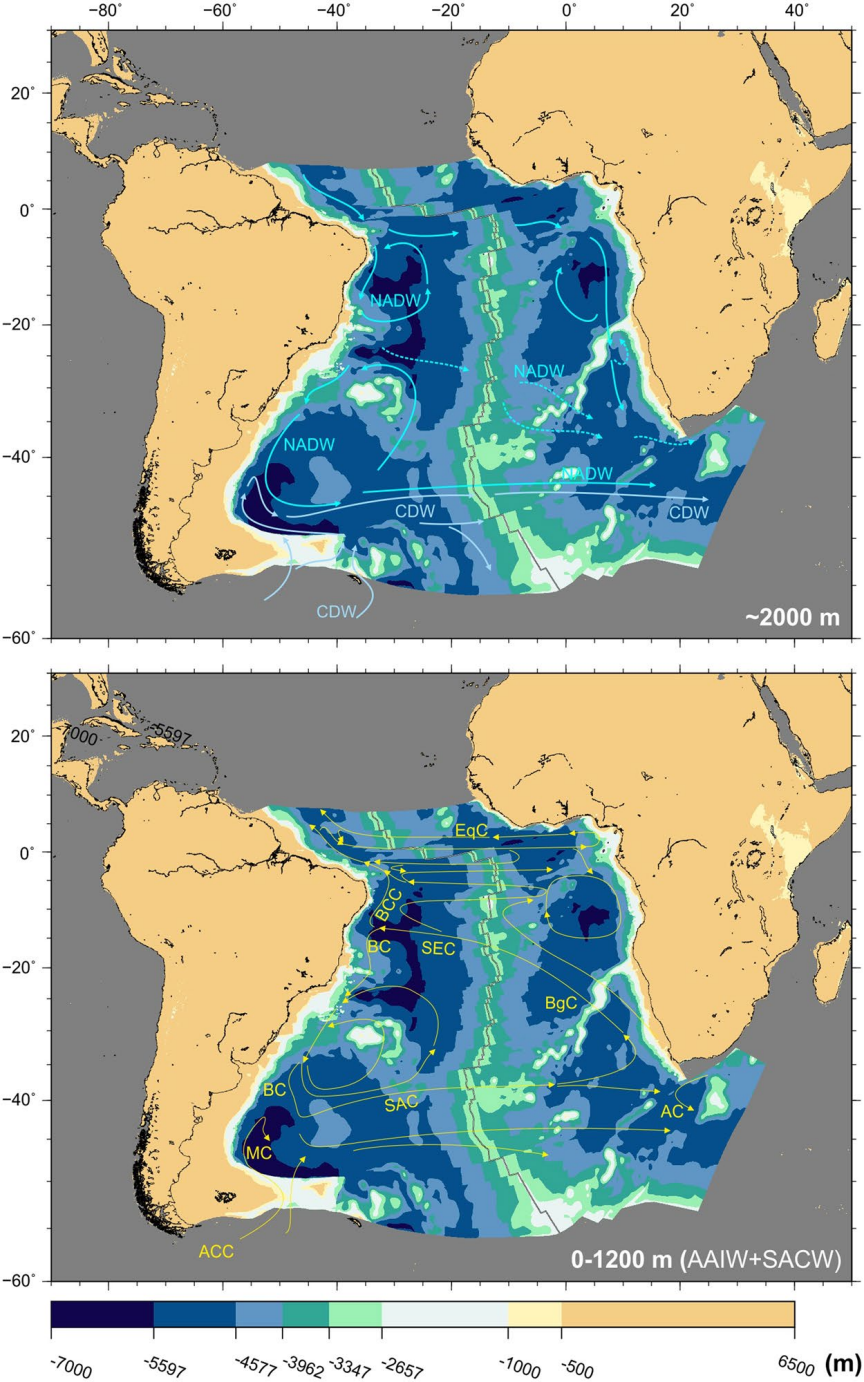
Modelled bathymetry at present-day and simplified patterns of intermediate and shallow water circulation. Water circulation paths are qualitative and arrow length does not indicate current speed. Top panel: Patterns of circulation of North Atlantic Deep Water (NADW) and Circumpolar Deep Water (CDW). Bottom panel: Patterns of circulation of Antarctic Intermediate Water and South Atlantic Deep Water. Both water masses circulate together as part of the following - AC: Agulhas Current; ACC: Antarctic Circumpolar Current; BC: Brazil Current; BCC: Intermediate Brazil Contour Current; BgC: Benguela Current; EqC: Equatorial Currents; MC: Malvinas Current; SAC: South Atlantic Current^77–80^. The map was generated using the Generic Mapping Tools^76^.

By the end of the Miocene, all elements of the present-day South Atlantic circulation system had formed. Bottom circulation was dominated by the Antarctic Bottom Water (Fig. 10). This water mass entered the Argentine Basin at depths greater than 3000 m, circulating (1) north along the western edge of the basin and (2) into the Argentine Basin generating an anticyclonic gyre. Only a small portion is able to travel across Rio Grande Rise and into the Brazil Basin. Intermediate depth circulation is dominated by the North Atlantic Deep Water, Circumpolar Deep Water and Antarctic Intermediate Water, the latter of which has been suggested to result from the interleaving of warm North Atlantic Deep Water with cold Antarctic Bottom Water south of the Rio Grande Rise^71^ (Fig. 11, top panel).

The circulation patterns of intermediate waters are differentiated north and south of the Rio Grande Rise. In the south, circulation patterns are controlled by the recirculation of the Antarctic Intermediate Water and South Atlantic Central Water and northward flow of Upper and Lower Circumpolar Deep Waters^72^. North of Rio Grande, both South Atlantic Central Water and Antarctic Intermediate Water flow northward as part of the Intermediate Brazil Contour Current (Fig. 11, bottom panel).

Surface water masses include the South Atlantic Central Water and Tropical Water, flowing south as part of the Brazil current, the western limb of the South Atlantic subtropical gyre. This current carries warm and salty waters towards the pole along the margin of South America and collides at the Brazil/Malvinas confluence with the Malvinas Current, which transports cold subantarctic waters towards higher latitudes^73^ (Fig. 11, bottom panel).

## Closing Remarks

The paleobathymetric reconstructions presented here represent a big step forward from previous attempts at modelling South Atlantic depth through time^2,5,74^. Because the uncertainties are better understood^8^, the results of the modelling process are a suitable framework in which to more confidently interpret the role played by tectonics in paleocirculation trends, as follows:

- Until the opening of an equatorial gateway at 100 Ma, circulation of water into the South Atlantic was controlled by the topography of the Falkland Plateau and early surface products of the Bouvet plume. Water entering the South Atlantic would have initially done so via the Agulhas Gap.
- As seafloor spreading progressed northward and the continental margins of Brazil and Angola started separating, a restricted basin formed north of the early Rio Grande-Walvis system. Limited circulation due to the topography of this pair of volcanic lineaments favoured a very restricted environment in this basin, leading to the deposition of thick evaporite layers.
- By 100 Ma, the Falkland Plateau cleared the Cape region of Africa, facilitating the entry of southern water into the South Atlantic. Furthermore, a shallow and intermediate connection was established with the Central Atlantic, improving ventilation of the Brazil and Angola basins. North-south water exchange was restricted by the Rio Grande Rise and Walvis Ridge. Fully open conditions (>3000 m) across the equatorial gateway may have existed since the start of the Coniacian (90 Ma).
- Formation of the Vema Gap across the Rio Grande Rise started allowing water exchange between the Argentine and Brazil basins in the Maastrichtian (70 Ma). Conversely, anoxic conditions prevailed in the Angola Basin which, until the start of the Paleogene was confined on all sides by the Cameroon Volcanic Line, the Walvis Ridge, the mid-ocean ridge and the African margin.
- The opening of the equatorial gateway, which contributed to the establishment of connections between all major oceanic basins, may have been a key event in triggering the climatic changes experienced globally during the late Cretaceous.
- Widening and deepening of the South Atlantic and subsidence of the Rio Grande and Walvis pair continued during the Paleogene. The Brazil and Argentine basins became well connected via the Vema and Hunter gaps. However, in the eastern flank of the South Atlantic, water circulation across the Walvis Ridge remained limited until mid-Eocene (40 Ma).
- Deep water exchange between the Argentine and Brazil basins became possible in the mid-Miocene (15 Ma). No such connection exists across the Walvis Ridge.
- Paleobathymetric reconstructions for Neogene times show many similarities with the present-day South Atlantic.
- The feedbacks between climate, ocean dynamics and paleogeography are still only partly understood. The results we show here agree well with recent suggestions that paleogeography is the main controlling factor for water mass formation and circulation^1^. Sedimentological, faunal and geochemical studies investigating water circulation as well as paleoclimate modelling would greatly benefit from inputs from paleobathymetric models such as the one presented here.

## Electronic supplementary material

Supplementary Information
