# Supplementary material for: South Atlantic paleobathymetry since early Cretaceous
Source: Sci Rep. 2017 Sep 18;7:11819. doi: 10.1038/s41598-017-11959-7 (PMC5603583; doi:10.1038/s41598-017-11959-7)
Supplement: Supplementary file 1 — Supplementary Information [file 41598_2017_11959_MOESM1_ESM.pdf]

South Atlantic paleobathymetry since early Cretaceous

Lucía Pérez-Díaz and Graeme Eagles

Supplementary Figures

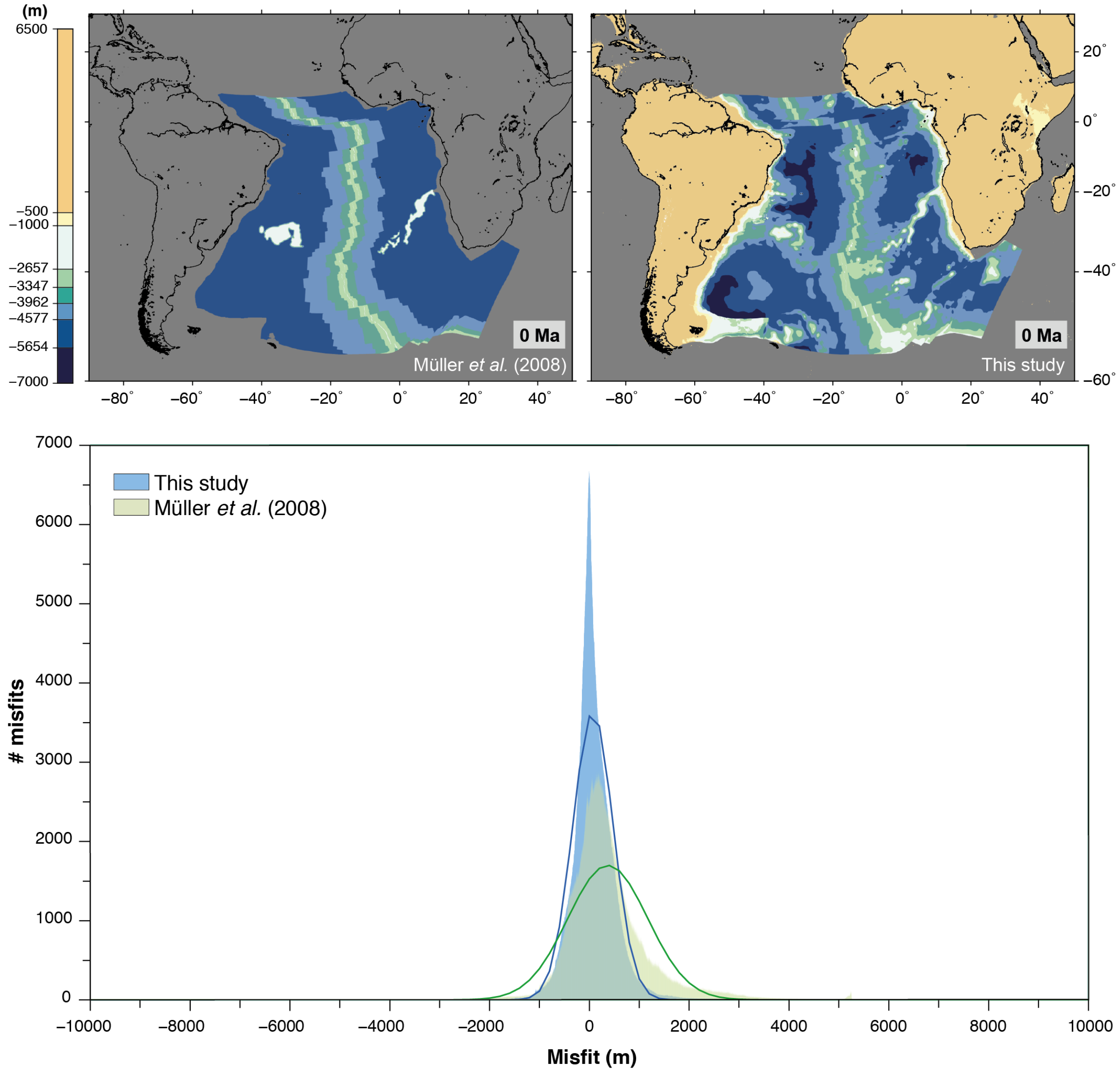

FS01: *Top left panel: present day bathymetry as modelled by Muller et al (2008). Top right panel: present day bathymetry as modelled in this study. Bottom panel: Misfits between the two present-day modelled bathymetries and satellite-derived present-day bathymetry. The maps in this figure were generated using the Generic Mapping Tools (Wessel, P. et al. 2013).*

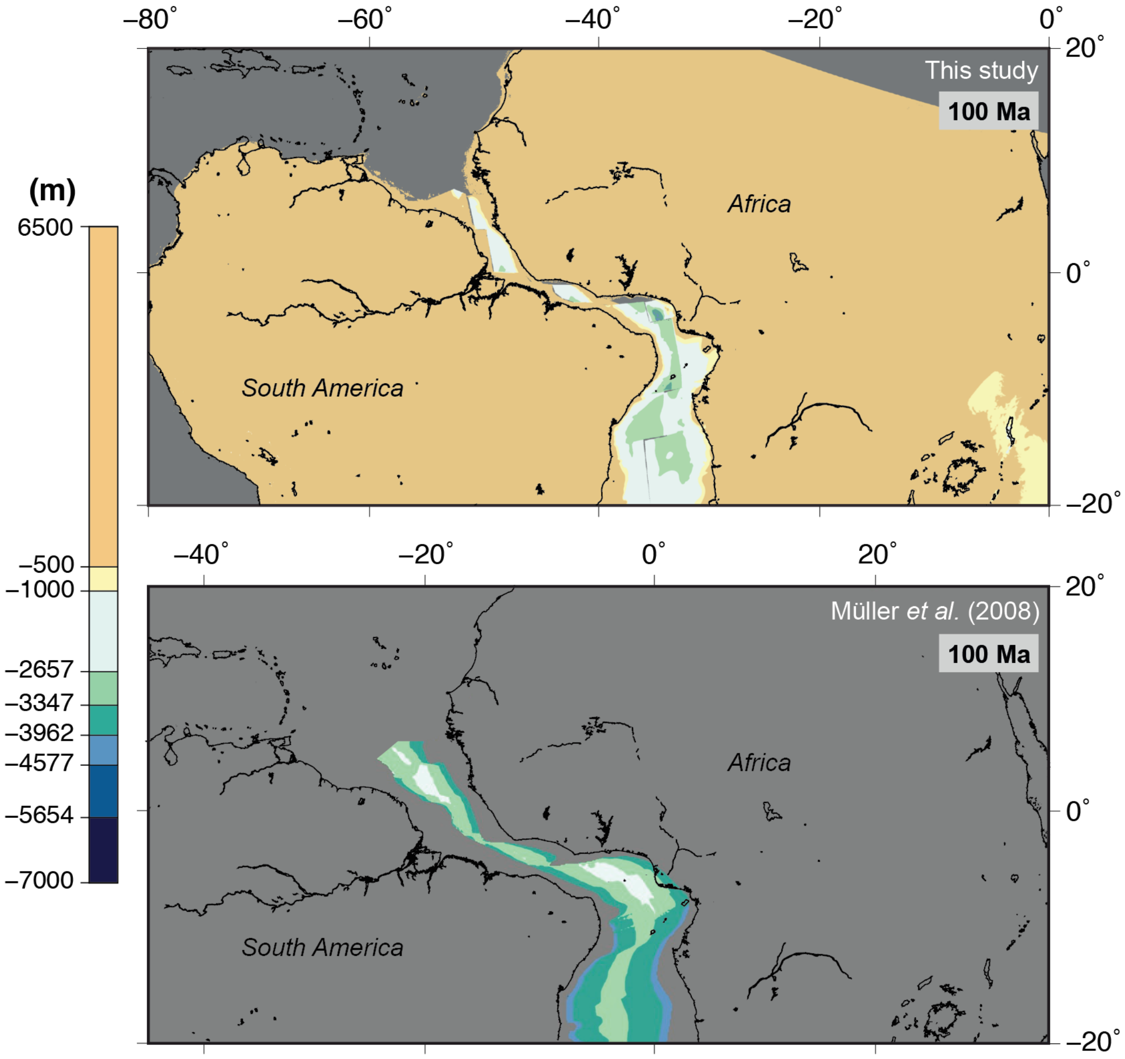

FS02: *Differences between modelled paleobathymetry at 100 Ma following our approach (top panel) and that of Muller et al. (2008) (bottom panel). The maps in this figure were generated using the Generic Mapping Tools (Wessel, P. et al. 2013).*
